# Supplementary material for: Mechanisms governing the pioneering and redistribution capabilities of the non-classical pioneer PU.1
Source: Nat Commun. 2020 Jan 21;11:402. doi: 10.1038/s41467-019-13960-2 (PMC6972792; doi:10.1038/s41467-019-13960-2)
Supplement: Supplementary file 7 — Source data [file 41467_2019_13960_MOESM7_ESM.zip › Source_Data/FigureS5/FigureS5A_MotifsCommonPeaks/homerResults/motif15.similar.html]

motif15

## Information for motif15

G
T
A
C
G
T
A
C
C
G
A
T
A
G
T
C
A
G
T
C
A
G
T
C
G
T
A
C
A
G
T
C
C
T
G
A
A
T
G
C
  
Reverse Opposite:  

T
A
C
G
G
A
C
T
A
C
T
G
A
C
T
G
C
T
A
G
A
C
T
G
A
C
T
G
G
C
T
A
C
A
T
G
C
A
T
G
  

|  |  |
| --- | --- |
| p-value: | 1e-111 |
| log p-value: | -2.561e+02 |
| Information Content per bp: | 1.710 |
| Number of Target Sequences with motif | 1963.0 |
| Percentage of Target Sequences with motif | 22.09% |
| Number of Background Sequences with motif | 4710.3 |
| Percentage of Background Sequences with motif | 12.39% |
| Average Position of motif in Targets | 100.4 +/- 53.5bp |
| Average Position of motif in Background | 99.4 +/- 63.8bp |
| Strand Bias (log2 ratio + to - strand density) | 0.1 |
| Multiplicity (# of sites on avg that occur together) | 1.13 |
| Motif File: | file (matrix) reverse opposite |

### Similar de novo motifs found

|  |  |  |  |  |  |  |  |
| --- | --- | --- | --- | --- | --- | --- | --- |
| Rank | Match Score | Redundant Motif | P-value | log P-value | % of Targets | % of Background | Motif file |
| 1 | 0.751 | T C G A C T A G C T A G C T A G T C A G C T A G G C T A T A C G A C T G G T C A | 1e-103 | -238.926341 | 12.41% | 5.49% | motif file (matrix) |
| 2 | 0.975 | G T A C G A T C C G A T A G T C G T A C A G T C G T A C A G T C C T G A A G T C G T C A | 1e-103 | -237.509317 | 18.48% | 9.90% | motif file (matrix) |
| 3 | 0.788 | A C T G A C T G A T C G A T C G A T C G T C A G A T C G | 1e-64 | -149.656756 | 34.00% | 24.92% | motif file (matrix) |
| 4 | 0.932 | G A T C G A T C C A G T A G T C G T A C G A T C G T A C A G T C C T G A T A G C G T C A G A C T | 1e-54 | -126.533056 | 9.98% | 5.27% | motif file (matrix) |
| 5 | 0.784 | A C T G A C T G A C T G A T C G A T C G T C A G A C T G A C T G | 1e-51 | -118.181038 | 20.19% | 13.63% | motif file (matrix) |
